# A unified framework for evolutionary genetic and physiological theories of aging

**DOI:** 10.1371/journal.pbio.3002513

**Published:** 2024-02-27

**Authors:** Jean-François Lemaître, Jacob Moorad, Jean-Michel Gaillard, Alexei A. Maklakov, Daniel H. Nussey

**Affiliations:** 1 Université Lyon 1, CNRS, Laboratoire de Biométrie et Biologie Évolutive UMR 5558, Villeurbanne, France; 2 Institute of Ecology & Evolution, School of Biological Science, University of Edinburgh, Edinburgh, United Kingdom; 3 School of Biological Sciences, University of East Anglia, Norwich, United Kingdom

## Abstract

Why and how we age are 2 intertwined questions that have fascinated scientists for many decades. However, attempts to answer these questions remain compartmentalized, preventing a comprehensive understanding of the aging process. We argue that the current lack of knowledge about the evolution of aging mechanisms is due to a lack of clarity regarding evolutionary theories of aging that explicitly involve physiological processes: the disposable soma theory (DST) and the developmental theory of aging (DTA). In this Essay, we propose a new hierarchical model linking genes to vital rates, enabling us to critically reevaluate the DST and DTA in terms of their relationship to evolutionary genetic theories of aging (mutation accumulation (MA) and antagonistic pleiotropy (AP)). We also demonstrate how these 2 theories can be incorporated in a unified hierarchical framework. The new framework will help to generate testable hypotheses of how the hallmarks of aging are shaped by natural selection.

## Abstract 2

Why and how we age are two intertwined questions that have fascinated scientists for many decades. This Essay proposes a new hierarchical model that links genes to vital rates, enabling a critical re-evaluation of the disposable soma theory and the developmental theory of aging in terms of their relationship to evolutionary genetic theories of aging.

## Introduction

Our understanding of the physiological and cellular mechanisms underpinning the aging process has been revolutionized over the last few decades with the emergence of a maturing field of biogerontology armed with diverse model organisms, experimental tools, and powerful “omic” approaches [1–4]. This has culminated in a growing consensus over key molecular and cellular processes that regulate aging and lifespan across distantly related model organisms [4–6]. These interdependent processes, called “hallmarks of aging” [7,8], constitute a list of prime candidate cellular mechanisms of aging that might translate to interventions to improve human healthspan [8]. The hallmarks represent a useful way to organize our current understanding of the mechanisms of aging and guide future experimental studies.

However, it has been argued recently that the hallmarks do not offer a predictive framework for understanding variation in the aging process [9]. Aging (or senescence) is a profoundly complex biological process for which both timing and intensity vary across species [10,11], populations [12,13], and individuals [14,15], as well as among cells, tissues, and organs [8]. Testable hypotheses that predict how these hallmarks account for the striking diversity in aging patterns that exists between and among species are still lacking [9], and studies tackling these questions fail to simultaneously incorporate evolutionary and mechanistic insights [16,17].

The emergence and establishment of an evolutionary theory of aging (ETA) long predates the emergence of modern biogerontology [18–21]. The ETA rests on the key tenet that the force of natural selection must decrease with increasing age. Vital rates—age-specific rates of survival and fecundity—underpin the Darwinian fitness of individuals within a population. The ETA demonstrates that selection acting on vital rates declines with increasing age [20]. While biogerontologists seek to understand the mechanisms underpinning the aging process, ETA models generally map alleles directly onto vital rates and thus say little about the cellular and physiological processes and pathways responsible for aging. Yet, 2 theories explicitly link evolutionary forces with the physiological mechanisms of aging: the “disposable soma theory” (DST) [22,23] and the “developmental theory of aging” (DTA) [24–26]. However, the relationship between these evolutionary physiological theories of aging and the evolutionary genetic theories remains unclear, and the links with the newly emerged hallmarks of aging are rarely discussed. To fill this gap, we propose a hierarchical evolutionary framework that integrates physiological and genetic theories to improve our understanding of the biology of aging.

## Evolutionary theory of aging: A hierarchical framework

The ETA is rooted in the decline of natural selection with increasing age. This means that alleles with deleterious effects in late life are more likely to escape selection and persist in the so-called “selection shadow” cast on later adulthood [18]. Hamilton [20] famously and axiomatically framed this in mathematical terms, showing how selection against age-specific mortality and for age-specific fecundity must decline with increasing age in age-structured populations. The pattern of this decline in selection’s strength depends on the populations’ mean vital rates, and the attenuation rate for selection is expected to shape the evolution of aging. Subsequent work has developed the theory by introducing specific population genetic models that elaborate on genetic aspects (e.g., mutation rates, pleiotropy across ages; [21,26]) or on how the number of mutations scale to the vital rates [27]. However, the central insight that selection should weaken with age remains unchallenged in age-structured populations, and the association between changes in survival and fecundity schedules and changes in individual Darwinian fitness is at the core of the evolutionary genetic theory of aging.

Two candidate evolutionary processes are generally considered in the ETA. The “mutation accumulation” (MA) model [18] assumes that germ-line mutations with deleterious age-specific effects on vital rates constantly emerge as genes are transmitted from one generation to the next. Natural selection will act to remove mutations that are deleterious to fitness. However, because the strength of selection weakens to become negligible at old ages, mutations with deleterious effects on late life only can accumulate under “mutation–selection balance.” Under MA, aging evolves because mutations have age-specific effects (i.e., different effects at different ages) and because selection is too weak to eliminate mutations whose deleterious effects are concentrated late in life. The “antagonistic pleiotropy” (AP) model [19] proposes that alleles with opposing effects on fitness in early and late life underpin the evolution of aging. As selection is strongest in early life, alleles with beneficial early-life effects but detrimental late-life effects should be favored. Likewise, alleles with detrimental effects on fitness in early life should be removed by selection, even if they are beneficial later in life. Two key differences exist between the AP and the MA models. First, AP assumes alleles have opposing effects on fitness in early and late life, while MA (in its simplest form) assumes detrimental alleles have effects on fitness at any age. Second, AP models invoke “purifying” or “balancing” selection processes, with selection acting to favor or fix alleles that enhance early life fitness at a larger extent than they decrease fitness later in life, while MA models invoke mutation–selection balance processes under which late-acting alleles can persist and accumulate due to the increasingly weaker selection with increasing age. These 2 models are in no way mutually exclusive: alleles with AP effects can co-occur with alleles that have consistently beneficial or detrimental effects at all ages.

**Fig 1:**
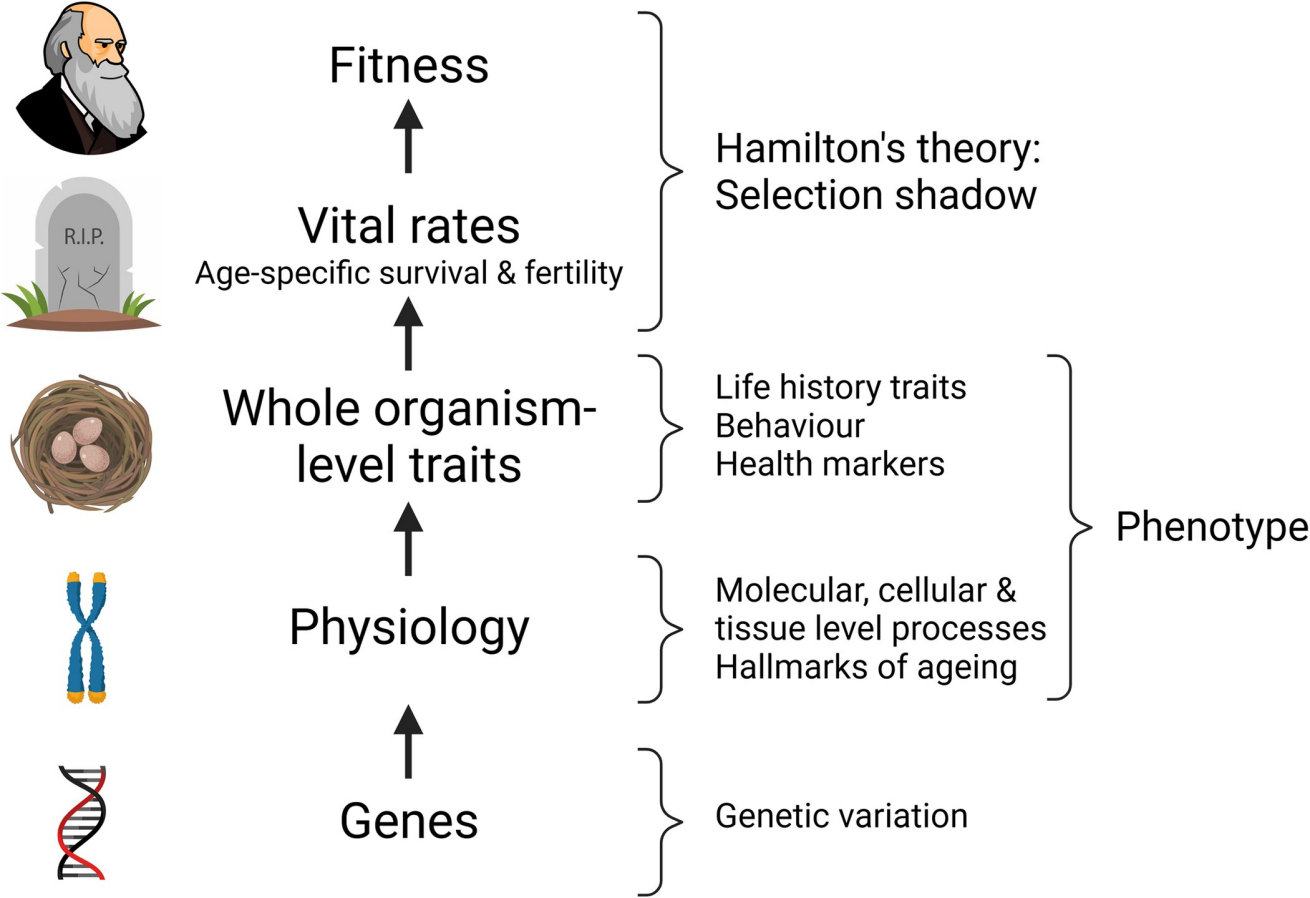
A biological hierarchy framework for understanding the causes of variation in aging. Fitness is wholly determined by vital rates, and it is the deterioration of these vital rates with increasing age that defines demographic senescence (actuarial senescence/aging is an age-related increase in mortality rates and reproductive senescence/aging is an age-related decline in reproduction). Underneath this we place the “phenotype,” which encompasses 2 levels of biological processes. “Whole organism-level traits” reflect aspects of function at the organismal level and can include life history, behaviors, morphology, or health. These traits are often the focal point of studies in evolutionary ecology, but they may also encompass health or morbidity outcomes in biogerontological or biomedical studies. Many such traits are known to deteriorate over the life course and may or may not contribute to demographic senescence. “Physiology” encompasses a complex array of molecular, cellular, and tissue level processes. Here, the work of biogerontologists to establish candidate hallmarks of aging can help us focus research efforts. At the same time, placing hallmarks of aging in a wider biological context can help us understand how they contribute to variation in whole organism aging and why they have evolved. At the base of the hierarchy sit “genes”: the number and states of difference alleles underlie the heritable nature of this framework and thereby permit evolution across all levels. Image created in BioRender.com.

These population genetic models of aging offer limited insight into the cellular and physiological processes that underpin variation in the way individual traits associated with behavior, morphology, life history, or demography decline with increasing age. However, as the field of aging will undoubtedly benefit from integrative approaches exploring the hallmarks of aging through an evolutionary biology lens, we must look beyond genes and vital rates [26,28]. We thus propose a hierarchical framework, similar to that used by Finch and Rose [29] and closely related to a model proposed by Moorad and Ravindran [30]. This hierarchy builds from genes at the lowest level of biological organization to vital rates and fitness at the highest level (as in the ETA) and incorporates various intermediate levels. We propose 2 candidate intermediate levels in Fig 1, which are intended to capture hallmarks of aging of primary interest to biogerontologists (physiological processes) that give rise to whole organism traits of interest to fields like evolutionary ecology, epidemiology, and medicine. For simplicity, we refer to these combined intermediate levels between genes and vital rates as “phenotypes.” Two evolutionary theories of aging (DST and DTA) offer promising insights on the way that natural selection may have shaped the age-specific changes at the phenotypic levels. We discuss the central concepts behind both physiological theories and relate them back to the genetic theories of aging that preceded them. We show how these models fit within our evolutionary hierarchical framework to allow phenotypic variation to be explored within a general ETA.

## The disposable soma theory

The DST is perhaps the first conceptual attempt to merge evolutionary thinking and proximate mechanisms into a single general mechanism for aging. In its original formulation, this theory presents aging as “an energy-saving strategy of reduced error regulation in somatic cells” [22]. More specifically, Kirkwood hypothesized that the broader manifestations of aging in multicellular organisms resulted from failures in the replicative potential of cells via errors in the synthesis of DNA and other macromolecules [22]. He then drew a distinction between “somatic cells which do not contribute information to succeeding generations and germ line cells which do” [22]. Kirkwood argued that the ability of an organism to maintain replicative accuracy in the germ line is essential to fitness and should be under strong selection. However, maintaining a high enough level of replicative accuracy in somatic cells to prevent organismal aging might be “a luxury our genes do better to forego,” because the energetic costs of maintaining fidelity of cellular replication and repair have to be weighed against the use of limited energy and time for other processes associated with fitness (e.g., growth and reproduction [22,31]).

During the 1980s, the DST was progressively re-framed in terms of resource allocation trade-offs [23,31]. As stated by Kirkwood, “the necessity for trade-offs arises because resources allocated to one function are not available to another” [32], which constitutes a redefinition of the principle of allocation initially defined by Cody [33]. This principle constitutes a central tenet of life history theory: limited availability or ability to acquire resources from the environment creates an allocation trade-off between major biological functions underpinning fitness, especially growth, reproduction, and survival [33,34]. This principle of allocation does not consider any specific molecular mechanism and does not rely on distinction between soma and germline.

The DST is widely accepted to rest on 2 central tenets. First, aging is the result of damage accumulation, an idea that rapidly became predominant in the literature on aging during the 20th century [35,36]. In the original formulation of the DST [22], damage accumulation stems from the “error catastrophe” theory [37], which posits that molecular damage resulting from stochastic errors that inevitably occur during biosynthesis stages (e.g., DNA transcription or replication) increases exponentially with age [22,37]. Newly arising damage interacts with and exacerbates existing damage to drive this accumulation. We note, however, that damage accumulation can arise independently of resource limitation and associated allocation trade-offs and, therefore, this assumption is not unique to the DST. Second, the mechanisms that have evolved to repair and protect against molecular damage (so-called “somatic maintenance” functions) are energetically demanding and, for a given and limited amount of resources available, allocating resources to maintenance reduces allocation to reproduction [22,31]. “Maintenance” captures a potentially very broad spectrum of biological processes, including the development of nondurable body parts (e.g., teeth), cellular renewal (e.g., replacement of skin cells), and intracellular maintenance (e.g., DNA repair) [31]. The resource allocation trade-off between costly maintenance, growth, and reproduction is a necessary condition of the DST, leading the limitation of resources to be a prerequisite of the DST.

The DST acknowledges that the aging process evolves through natural selection [22,23]. Imperfect maintenance of cells allows damage to accumulate and ultimately results in the deterioration of physiological function in later life, where the strength of selection for that function is expected to be weakest. As a result, imperfect somatic maintenance and aging are expected to evolve as selection favors allocation towards reproduction over indefinite maintenance [31]. As an evolutionary theory, the DST thus falls under the umbrella of the AP model [26,31]. Yet, despite this clear link, the DST and AP are often presented as alternative theories in the aging literature [38]. Various arguments developed so far to justify distinction between the DST and AP include the presence of a distinct soma and germline [39], polygenic regulation of aging [40], concomitant effects of genes boosting reproduction on damage accumulation rather than delayed effects [39], and natural selection acting on maintenance rather than reproduction [32,39]. None of these seem to us to be incompatible with the broad framework offered by the AP model and we therefore argue that the DST is a physiologically explicit case of AP, a view already expressed in the literature [4,41,42]. Specifically, the DST hypothesizes that variation in age-specific vital rates is underpinned by resource allocation trade-offs between maintenance functions and other fitness-related traits and processes driving them and requires resource limitation in the environment (see also [26,41–45]; Table 1).

**Table 1:** A summary of key assumptions and predictions of the DST and the DTA.

	Disposable soma theory	Developmental theory of aging	
		Hyperfunction	Hypofunction
**Genetic evolutionary model**	Antagonistic pleiotropy	Antagonistic pleiotropy or mutation accumulation	
**Role of resource limitation**	Aging driven by insufficient allocation of limited resources to maintenance functions	Resource limitation or allocation are explicitly not invoked	
**Proximate cause of aging**	Damage due to insufficient maintenance	Excessive function of gene involved in early life fitness	Insufficient function of gene involved in early life fitness
**Key distinct predictions**	Diverting resources to maintenance postpones the onset of aging and/or slows down the aging rate at the cost to reproduction	Reduced function postpones the onset of aging and/or slows down the aging rate	Increased function postpones the onset of aging and/or slows down the aging rate

DST, disposable soma theory; DTA, developmental theory of aging.

**Fig 2:**
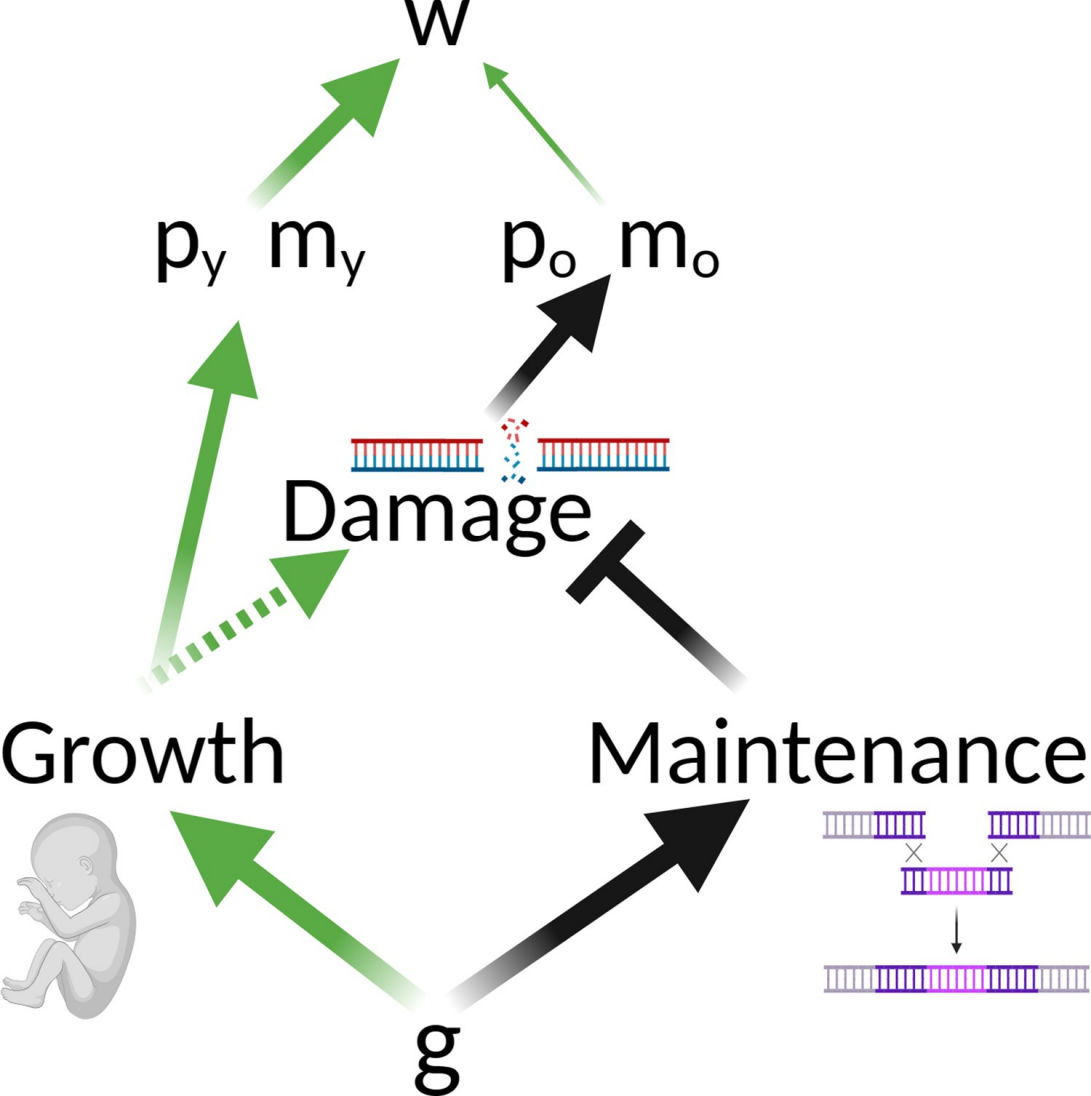
The DST of aging. In the figure, “g” reflects alleles that influence the allocation of limited resources towards growth and maintenance functions, p_y_ and m_y_ are vital rates (survival and fecundity, respectively) when young, and p_o_ and m_o_ are vital rates when old. Green arrows reflect positive effects, and black arrows indicate negative effects. The figure illustrates the effects of an allele which increases the proportional allocation of resources to growth (green arrow to growth), thereby reducing allocation to maintenance functions (black arrow to maintenance). This can be contrasted with an allele with the opposite effect of allocating proportionally less to growth and more to maintenance. Increased allocation to growth has positive effects on vital rates in early life (green arrows from growth to p_y_ and m_y_), but it may also indirectly cause more damage (dashed green arrow from growth to damage). This will result in senescence and reduced late-life vital rates (black arrows from damage to p_o_ and m_o_). Allocation to maintenance reduces or repairs damage (black inhibition line from maintenance to damage), and reduced allocation to maintenance will indirectly result in more damage and reduced vital rates in later life. The allele illustrated here could be favored by selection over one (or more) allele(s) which allocated more to maintenance because selection is stronger on early-life vital rates (width of arrows linking vital rates and fitness when young versus old). DST, disposable soma theory.

To clarify this point, we consider both AP and the DST within the hierarchical framework presented in Fig 1. AP models at their broadest relate age-specific gene action to vital rates and determine the likely evolutionary outcome. The DST is explicit about the biological processes that link genes and vital rates, and we illustrate this in Fig 2. For simplicity, we consider a pleiotropic gene that controls the allocation of resources between growth and maintenance in a population facing resource limitation. Increased allocation to growth results in improved early-life performance (green arrows from growth to early vital rates) through, for example, earlier or larger size at maturity, reduced environmentally driven risks of mortality, or earlier or greater fecundity. However, when resources are limited, this will also reduce allocation to maintenance, resulting in reduced late-life performance (black arrows from maintenance to late vital rates) such as an earlier onset and/or more rapid aging. Increased allocation to traits associated with early-life performance is typically thought to result in increased damage independently of the amount of resources available, which will exacerbate the detrimental effect on aging (dashed green line from growth to damage in Fig 2). However, aging would still be expected to evolve under this model without growth-associated damage purely due to reduced allocation to maintenance. As Fig 2 illustrates, the weakening of selection with increasing age (paths linking vital rates and fitness) means that selection is likely to favor some degree of reduced allocation to maintenance. This is because the fitness costs of accumulated damage in later life will be outweighed by the early-life fitness benefits of increased allocation to growth and reproduction. Under the DST, aging will vary due to differences in the rate of accumulation of molecular damage. The evolution of aging will be determined by the available genetic variation in resource allocation strategies and the relative fitness costs and benefits of each strategy given the patterns of declining selection with increasing age.

Genetic influences on growth and maintenance could be completely age-independent under the DST. In this case, the allocation strategy could be constant within a given life history stage (e.g., among adults). However, this would still result in an age-dependent genetic effect on the vital rates, which is a fundamental assumption of evolutionary theories of aging. The allele illustrated in Fig 2 will have positive effects on early-life vital rates but negative effects on late-life vital rates, despite potentially showing no age-specific effects on the allocation of resources to growth and maintenance during the growth period. Furthermore, because the net effects of genes shaping the evolution of aging under the DST have antagonistic effects on early- and late-life vital rates, they behave in a manner entirely consistent with the broader class of AP models.

## The developmental theory of aging

A second physiological theory of aging with an evolutionary basis is the DTA. This theory asserts that aging results from suboptimal regulation of age-specific gene expression in adulthood and thus emerges as a by-product of strongly selected and regulated developmental processes [24–26]. From a broad evolutionary perspective, the DTA views the evolution of aging as the result of weakening selection with age resulting in “physiological processes that are optimized for early-life development, growth, and reproduction and are not sufficiently optimized for late-life function” [26].

### Hyperfunction and hypofunction

The most widely discussed process through which the DTA might manifest is the continued expression of cellular pathways which favor growth and development, and thereby early-life fitness, but which become deleterious to fitness in late life and ultimately contribute to increased aging [24]. This is termed the “hyperfunction theory of aging” [46]. Blagosklonny [46] specifically associates the term with a particular pathway—the target of rapamycin (TOR) pathway—suppression of which extends lifespan and delays aging across distantly related laboratory model organisms [46,47]. He argues that TOR expression is essential in early life for development, growth, and anabolic cellular function but continued high levels of expression in later adulthood are associated with late-onset pathologies including osteoporosis and myocardial infarction in humans [46]. In his paper that introduces the AP model of aging, Williams illustrates with an example of a similar process. He proposes a gene promoting calcification that enhances bone growth in early life but, with continued expression, results in a loss of arterial elasticity and subsequently heart disease in late life [19].

**Fig 3:**
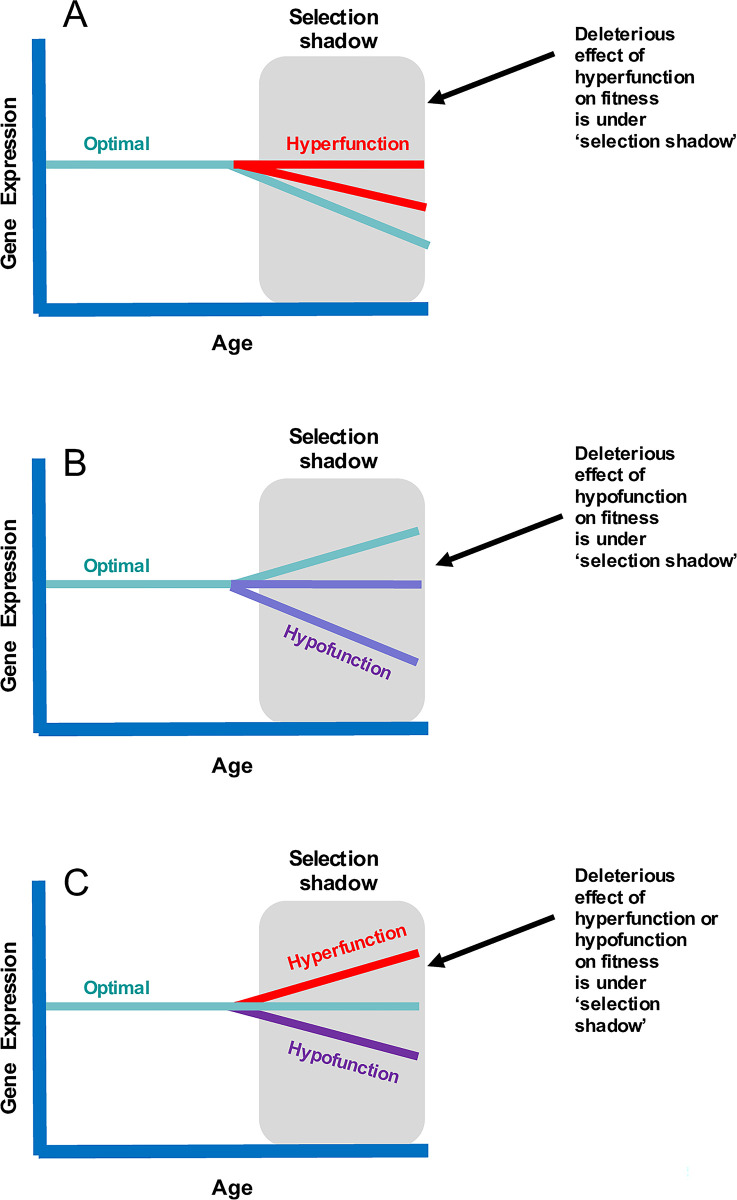
The DTA through hyperfunction and hypofunction pathways. The DTA suggests that selection does not sufficiently optimize age-specific biological function because the force of selection on traits declines with increasing age. The DTA covers a broad range of scenarios where the biological function is higher or lower than optimal for a given age. The “selection shadow” reflects late life, when selection is weak or absent under natural conditions. Three potential patterns are illustrated. First, consistent continuous biological function can become deleterious in late ages, but natural selection is too weak (“selection shadow”) to result in the evolution of a modifier gene that will down-regulate (or sufficiently down-regulate) late-life expression of the focal gene (hyperfunction; **A**). Second, it could be beneficial to increase biological function with age, but selection in late life is not strong enough to achieve this, and so expression evolves to be below the optimal level in late life (hypofunction; **B**). Finally, it could be optimal to maintain biological function at a constant level with age, but weak selection in late life results in misregulation and either over (hyperfunction) or under (hypofunction) expression in old age (**C**). DTA, developmental theory of aging.

Hyperfunction is just one potential way in which the DTA might manifest. Alternatively, the switching-off or suppression of a developmental pathway in late life or the failure to maintain expression at adequate level after development is complete could contribute to aging [24,26]. This mechanism has been termed “hypofunction” [26]. Putative examples include the rapid suppression of heat shock protein expression in worms during early adulthood that results in a weakened adult stress response [26,48], sarcopenia [24], or an absence of tooth replacement in late life in mammals such as elephants, where cycles of tooth rotation occur [49]. Fig 3 illustrates the different ways in which the DTA might manifest, clarifying that hyperfunction and hypofunction are 2 sides of this theoretical coin, depending on whether expression lies above or below the fitness optima in late life.

Recent verbal models of the DTA invoke direct effects of gene expression on function and fitness and frame the model as distinct from those invoking indirect effects of damage or repair processes [45]. We argue this is unnecessary from an evolutionary perspective. Aging could foreseeably evolve via DTA through direct, damage-independent effects on fitness (as envisaged in [24,45,46]). For example, non-optimized mammalian TOR pathway expression in late life could directly cause cellular dysfunction and disease. However, the DTA does not preclude a role for damage or failure of repair/replacement function in late life. For example, hypofunction could manifest through loss of proteostasis, a key hallmark of aging [7,8]. Genes controlling the production of molecular chaperones and protein degradation (ubiquitin–proteasome system and autophagy) may be optimally expressed in early life for development and fitness but insufficiently expressed to prevent the ultimate accumulation of misfolded proteins later in life. This could result in aging through indirect effects of a gene (or genes) optimized for early-life fitness but insufficiently expressed to prevent functional failure at some point in late life (Fig 3). Tissues or biological structures which have a fixed form or size once development is complete (e.g., tooth size in some mammals, wing or body structure in some insects) but show deterioration via environmental wear and tear may represent similar examples of hypofunction (e.g., tooth wear limiting feeding and wing damage limiting flight [50,51]). The key difference here with the DST lies in the critical fact that resource allocation trade-offs are not involved. A fundamental question for the DTA theory is whether it purely invokes direct effects of age-specific gene expression or whether it can involve indirect effects of damage accumulation as well. We would argue these are important mechanistic distinctions, but that the DTA currently encompasses both and can remain distinct from the DST as long as resource allocation trade-offs are precluded.

### Directional and optimizing selection

**Fig 4:**
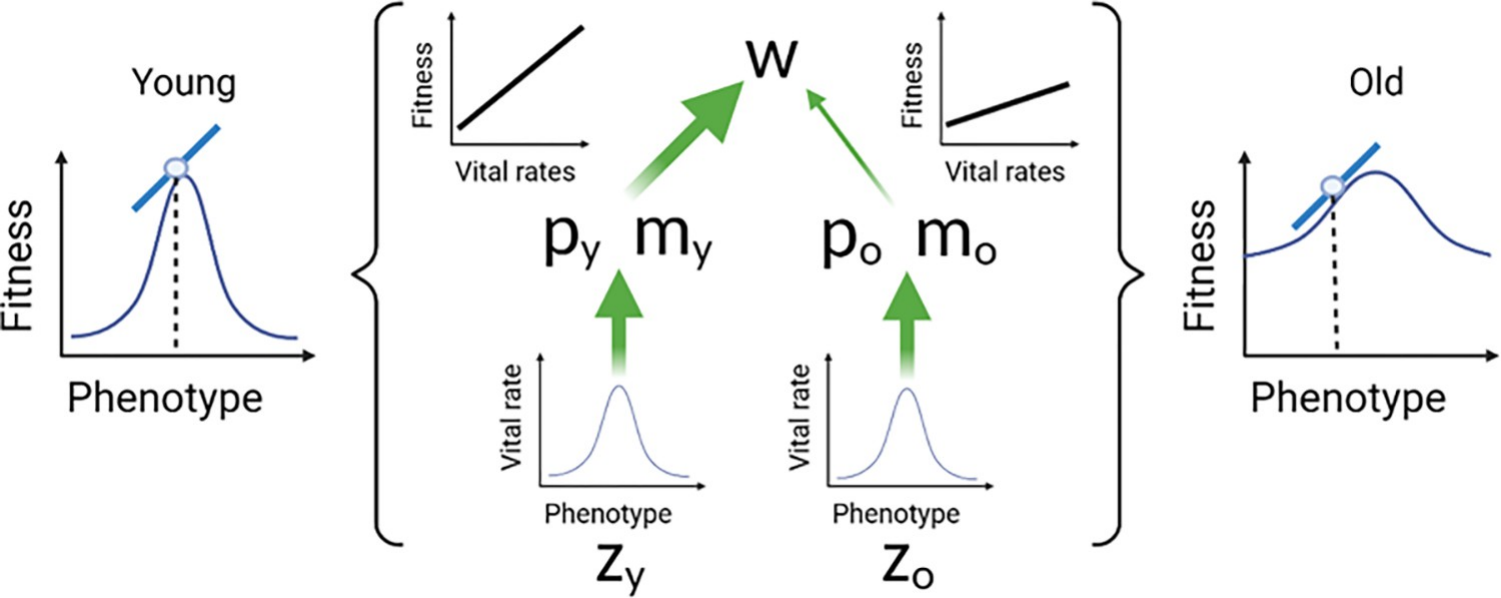
Directional and optimizing selection. This figure illustrates how the proposed optimality model that links phenotypes to vital rates, and captures verbal models of the DTA, fits into the hierarchy illustrated in Fig 1 and how it yields age-specific directional selection. The optimality functions (lower 2 plots in the center) are assumed to be identical at both ages. However, because selection emphasizes improvements to vital rates more in early life than at late life (upper 2 plots in the center), the sensitivity of fitness to changes in the early-age phenotypes (left) is greater than the fitness sensitivity to changes in the late-life phenotype (right). These outer functions define age-specific directional selection, which is the slope at the population-mean phenotype under our model assumptions. The blue circles represent these means, and the blue lines are the slopes at those points. Note that the same amounts of directional selection can exist at both ages, provided that the late-age phenotype is further removed from its optimum. DTA, developmental theory of aging.

Although increasingly discussed and tested in the biogerontological literature, the DTA has never been formally framed within the ETA. However, much like the DST, the DTA is has been viewed as an AP mechanism [25,26]. As we show below, however, the DTA can work via both MA and AP. Verbal models of DTA present aging as evolving due to a failure of optimization of key physiological processes by natural selection in late life. We can integrate this mechanism into the ETA by linking age-specific manifestations of these physiological processes (phenotypes) to vital rates at those same ages. The age-specific vital rates are then optimized at some specific phenotypic value; phenotypes that are below or above these values lead to lower vital rates. The phenotypic location of that vital rate optimum can itself be age specific. Let us also imagine that vital rate improvement diminishes as the phenotype approaches the optimum (a diminishing return); this can be visualized as a Gaussian function. The slope of this function at any point along the phenotypic axis quantifies the sensitivity of the vital rate to changes in the phenotype (see the 2 bottom, central functions in Fig 4).

These slopes can be interpreted in terms of natural selection by placing these functions into the context offered by the hierarchy presented in Fig 1. By multiplying the function described above (phenotype to vital rate) by the function that Hamilton used to describe selection for vital rates (fitness to vital rates), we get a new function that directly assesses the sensitivity of fitness to changes in the phenotype (Fig 4, outer functions). This is exactly a model of “optimizing selection” (*sensu* [52]), in which intermediate phenotypes have the highest fitness and extreme phenotypes (in either direction) have the lowest fitness. Optimizing selection is a description of the global function that relates fitness to the phenotype. The strength and direction of directional selection determines how the force of natural selection contributes to the change in the mean value of the phenotype over one generation. This is equal to the average slope of the global fitness function taken over all members of the population (which is equal to the slope at the mean phenotype when the distribution of individual trait values is Gaussian) [53]. Directional selection thus describes the slope of the local fitness function.

We can have 2 vital rates to phenotype functions that apply at 2 different ages but have identical shapes. However, because the “selection shadow” that leads to less selection at late ages applies, the fitness to late-age phenotype function will appear flatter, or more compressed along the fitness axis, than the corresponding fitness to early-age phenotype function (Fig 4). One consequence of this is that directional selection (the mean slope experienced by the population) will push the early phenotype more strongly towards the early-age optimum than the corresponding late phenotype, provided that they both are evaluated at the same distance from their optima. Second, diminishing fitness returns implied by the shape of the functions means that there will be early-life phenotypes close to their corresponding optimum that experience the same selection pressures as late-life phenotypes that are further from their optimum. As we argue below, this feature is important in determining aging rates that evolve through an MA process.

### Age-specific optimality selection

**Fig 5:**
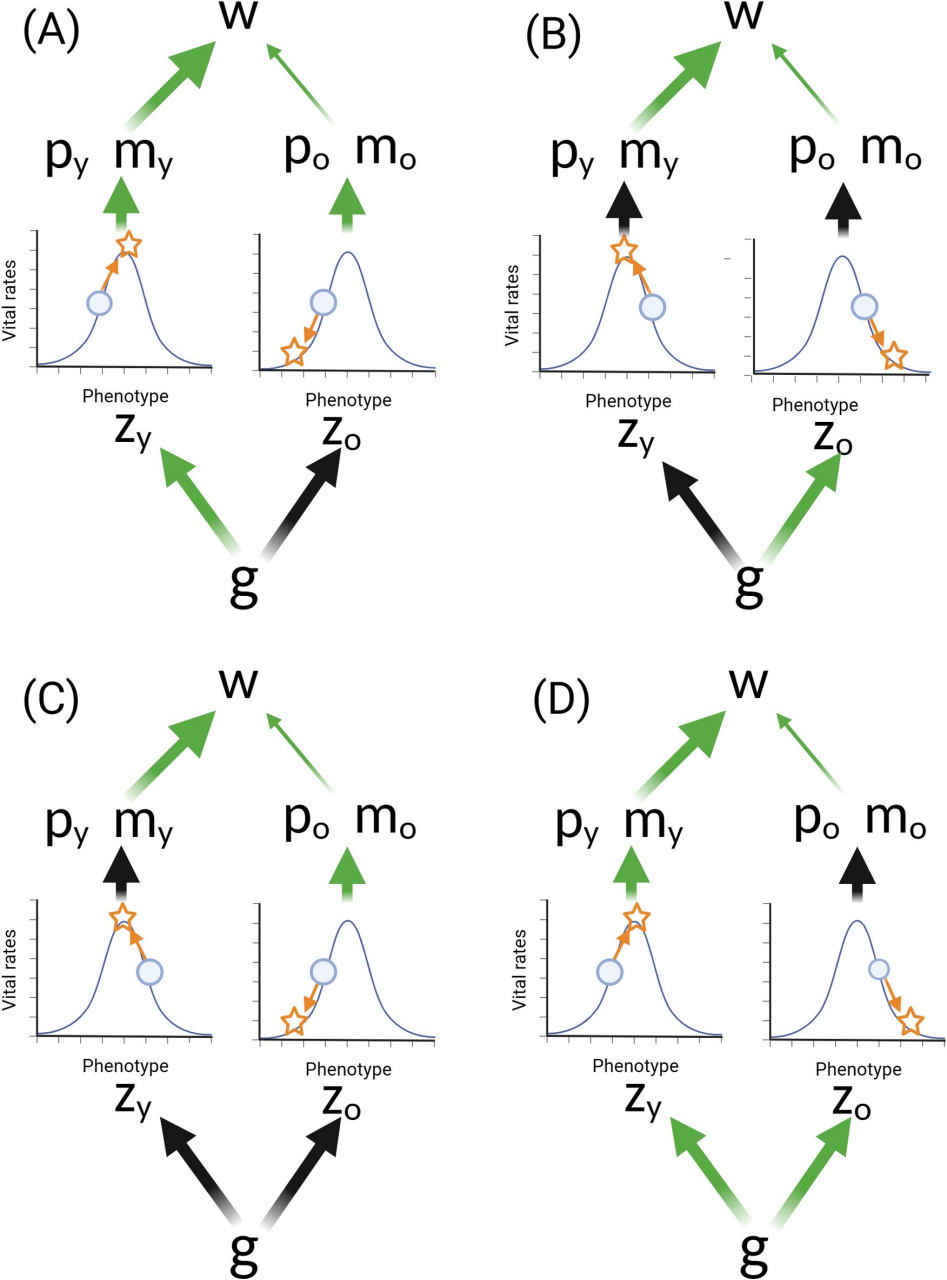
The DTA with age-dependent pleiotropy. Blue circles identify the phenotypic value of the population mean before senescence evolves, the orange stars identify its value afterwards, and “g” refers to a single allele that increases senescence. **A** and **B** illustrate cases where that allele has effects that are negatively associated across the age-specific phenotypes and the population mean phenotypes at early and late ages begin at suboptimal values that are on the same side of the optimum. (**A**) Hypofunction: the population mean phenotypes are less than their optimal values. Natural selection favors the increase of the trait early in life despite its costs to vital rates late in life. (**B**) Hyperfunction: the optima are below the initial population means. Selection favors a decrease in the early-life phenotype, and this causes an increase in the late phenotype. **C** and **D** illustrate cases where that allele has effects that are positively associated across the age-specific phenotypes and the population mean phenotypes at early and late ages begin at suboptimal values that are on opposite sides of the optimum. (**C**) Hypofunction: the population mean phenotype at early and late life are higher and lower than their respective optima, respectively. Natural selection will act to reduce early-life values, which causes late-life values to become lower and therefore more suboptimal. (**D**) Hyperfunction: the population mean phenotype at early and late life are lower and higher than their respective optima, respectively. Natural selection will act to increase early-life values, which causes late-life values to become higher and therefore more suboptimal. DTA, developmental theory of aging.

In the following discussion, we use a model of age-specific optimality selection to illustrate how hyperfunction and hypofunction can act through both MA and AP mechanisms to cause the evolution of aging. Here, we summarize these descriptions using the biological hierarchy introduced in Fig 1. In Fig 5, we present 2 scenarios through which this conceptualization of the DTA might lead to the evolution of aging when genetic effects on early and late life are negatively correlated (AP; Fig 5A and 5B). Interestingly, aging can also evolve under this model when genetic effects are strongly positively correlated and young and old phenotypes sit on opposite sides of their age-specific optima (Fig 5C and 5D). Whether hyperfunction or hypofunction evolves simply depends upon which sides of the peak the late-life phenotype ends up.

**Fig 6:**
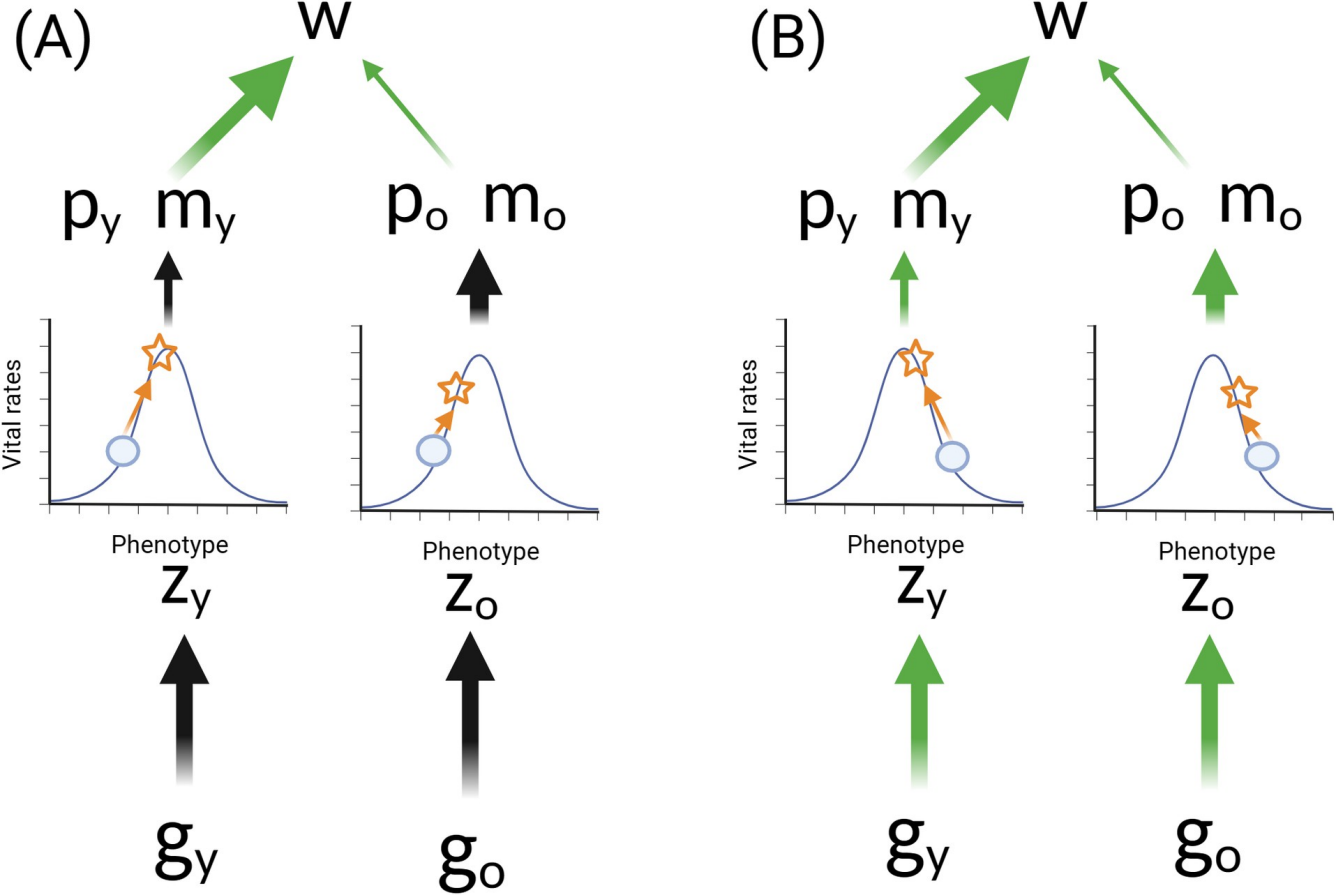
The DTA as an MA model. For the MA model, we imagine that there are 2 mutations with strictly age-specific effects (*g*_y_ and *g*_o_) that recur every generation with some equal and unspecified frequency. The joint effects of mutations and selection will cause the population to equilibrate with age-specific phenotypes that are closer to the early-age optimum than to the late-life optimum. If the effects of the mutations tend to reduce the phenotype, then hypofunction evolves (**A**). Alternatively, hyperfunction evolves if mutations tend to increase the phenotype (**B**). DTA, developmental theory of aging; MA, mutation accumulation.

Aging could also evolve in the DTA framework via MA processes (Fig 6A and 6B), contrary to the widely held view that the DTA is necessarily a specific case of the AP model. To illustrate this, we imagine that de novo mutations arise every generation and these mutations have age-specific effects on phenotypes that tend to move the mean phenotype in either a positive or negative direction. At the same time, directional selection will cause the population mean phenotypes to migrate up the vital rate gradient at both ages. We can expect that if the properties of these new mutations are the same for both ages (e.g., age-specific mutations tend to be equally damaging to vital rates at the different ages), then the populations will reach evolutionary equilibria at points where the early-age phenotype is closer to its vital rate optimum than the late-age phenotype is to its optimum. When the mean effects of new mutations on the phenotype are negative, then this senescence is described by hypofunction (Fig 6A), and when the mean effects are positive, then senescence via hyperfunction evolves (Fig 6B). This conceptualization of the DTA as age-specific optimizing selection is novel, but we feel it captures most verbal models of the theory in the literature adequately. It importantly presents the DTA as a process of weakened optimizing selection in late life, which could evolve through either AP or MA processes.

To illustrate these ideas in more depth, we begin by imagining that at every age, there is a standard Gaussian function that relates individual values of a phenotype to a vital rate manifested at that same age, and this vital rate is maximized at some intermediate phenotypic value (Fig 4). This age-specific optimal set of vital rates may change with age, but here we standardize it by assigning to each optimum a phenotypic value of “0.” Individual aging is indicated when age-specific values are at different distances from their respective means. “Senescence” is defined here to mean an age-related change that is deleterious with respect to vital rates. In this model, phenotypic senescence is found when the late-age phenotypic mean is further from its optimum in any direction than the early-age phenotypic mean is distant from its optimum. Likewise, equidistance between mean age-specific phenotypes and their corresponding optima equates to no aging, and negative senescence for that phenotype exists when the late-age phenotypic mean is closer to its optimum that its early age counterpart. This model suggests 2 forms of senescence associated with the DTA. Hypofunction implies that the late-acting phenotypic mean is further from its optimum because its value is too small, and hyperfunction follows when this mean is further from its optimum because it is too large. Below, we describe how this model causes phenotypes to evolve senescence from an initial non-aging state by the action of natural selection.

Let us assume for simplicity that the phenotype at a given age only affects a vital rate at that same age, and the distribution of individuals’ phenotypes within the population is also Gaussian. The “vital rate gradient” is the slope of this function found at the population mean phenotype. The strength of “directional selection,” or simply *β* that favors an increased phenotypic value is equal to the product of this vital rate gradient and the gradient that describes the strength of selection for the vital rate [30]. This latter gradient is independent of the phenotype and, as the ETA shows us, it weakens with age [20]. As we have assumed that the shape of the function that associates vital rates to trait values is the same across ages, it follows that the magnitude of *β* will be less at older age if phenotypes at the 2 ages are equidistant from their respective optima. However, it should also be noted that because the local curvature of the function experienced by the population changes with the mean phenotype, the vital rate gradient will tend towards zero as the population approaches the optimum. To summarize, directional selection for the age-specific phenotype, *β*, is the product of: directional selection for the vital rate, which must decrease with age, and the vital rate gradient, which must decrease as the population mean approaches its age-specific optimum.

A non-aging population will necessarily experience stronger *β* for the early phenotype because the vital rate gradient is defined to be identical for both age-specific phenotypes. Assuming equal amounts of heritable variation for the phenotypes at both ages, this difference in *β* will cause the evolution of incipient senescence because the early-age phenotypes will move more effectively towards its optimum than the late-age phenotypes. The same process will occur in the next generation, but because the early-age mean will begin closer to its optimum than the late-age mean, the vital rate gradient will become stronger for the late-age phenotype. This will reduce the difference between *β* at each age, and less new senescence will evolve in this generation. We can expect this process to continue to increase evolved senescence over subsequent generations until the population reaches an evolutionary equilibrium dictated by the genetic constraints specific to the 2 age-specific phenotypes. The nature of these genetic constraints differs between classical models of MA and AP, and we apply genetic models of both to the fitness functions implied by our optimization models in what follows.

**Fig 7:**
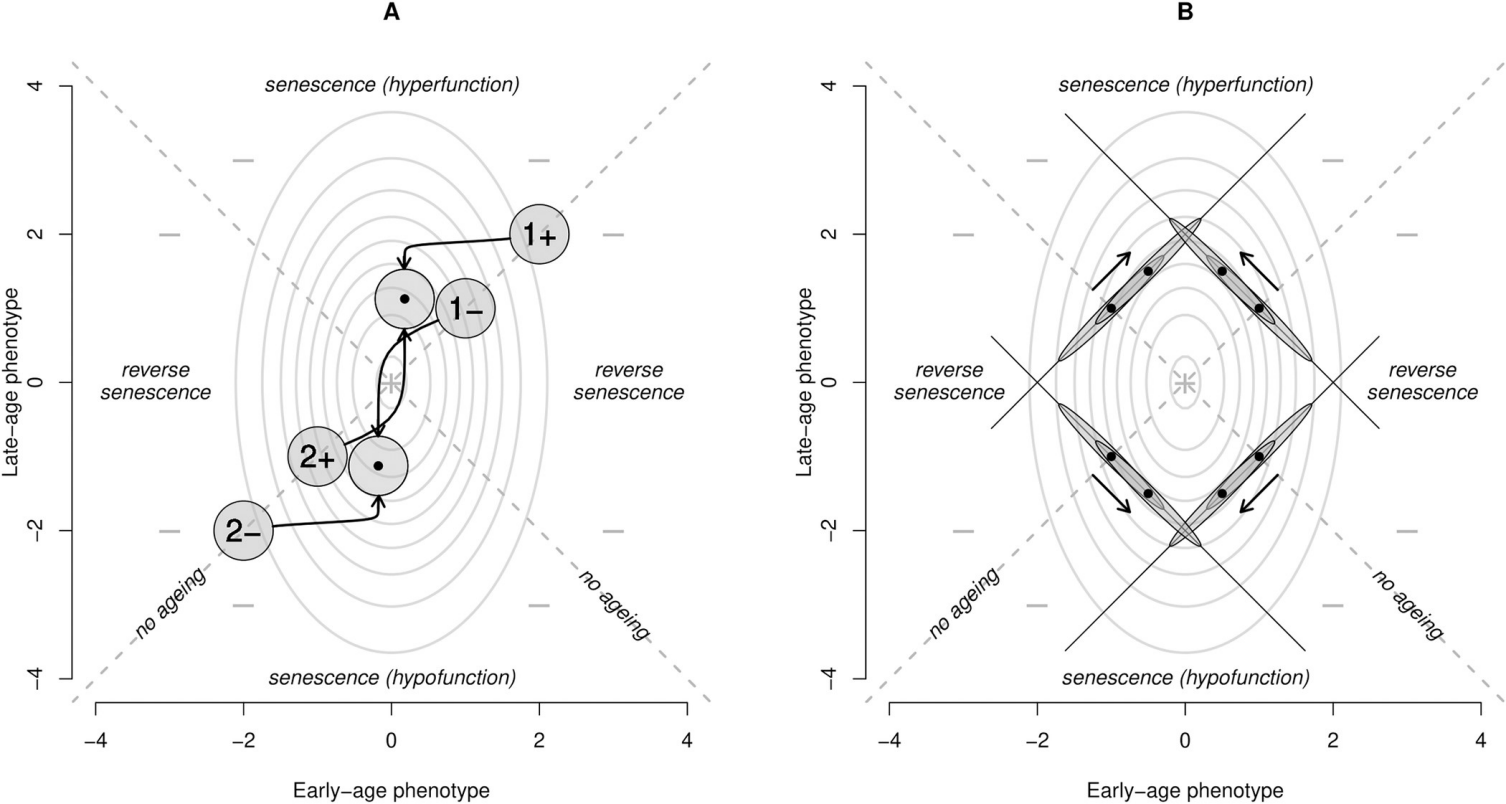
Reconciling the DTA and the ETA via optimizing selection. (**A**) Mutation accumulation. The contour plot illustrates the function that relates fitness (the contours) and early and late phenotype values (the *x* and *y* axes, respectively) when corresponding age-specific vital rates are optimized at intermediate phenotype values. This is a bivariate perspective of the univariate fitness functions illustrated in Fig 4. Negative values indicate regions of low fitness and the “+” identifies the phenotype combination that maximizes fitness. The *y* = *x* and *y* = -*x* axes define phenotypes that combine to describe no aging; the intersection of these axes creates quadrants of parameter space that describe different types of aging. Hyperfunction and hypofunction correspond to the top and bottom quadrants, respectively, and reverse senescence is indicated by phenotypes in the left and right quadrants. The distribution of phenotypes in a population is represented by gray circles, and the mean values are located at their centers. The arrows indicate the evolutionary progression of these populations from initial states defined by no aging to states consistent with senescence. (**B**) AP. The initial non-aging populations are represented by ellipses with bivariate means located on the *y* = *x* axis and indicated by points. The lines along the major axis of each identify the allowable phenotypic combinations that are permitted given the strict genetic constraints placed on the model. The arrows indicate the paths that these populations follow as they evolve towards the phenotype combinations with maximum allowable fitness. The final populations are described by the gray ellipses centered on this point. Senescence can evolve either with negative genetic correlations across phenotypes (the bottom-left and upper-right distributions that correspond to Fig 5A and 5B, respectively) or with positive genetic correlations across phenotypes (the bottom-right and upper-left distributions that correspond to Fig 5C and 5D, respectively). AP, antagonistic pleiotropy; DTA, developmental theory of aging; ETA, evolutionary theory of aging.

Age-dependent de novo mutations will arise in every new generation. In keeping with classical population genetic models of MA [54], we assume that mutations have strictly age-specific effects. Every generation, these mutations will change the mean age-specific phenotypes in some direction and magnitude that is constant over time (the mean mutational effect). We assume that this change is the same for both age-specific phenotypic means. We note that the direction of change in the mean phenotype can be positive or negative, and these directions can be shown to lead to hyperfunction and hypofunction, accordingly. To demonstrate, let us first change our perspective of age-specific vital rate to phenotype functions from the univariate view (Figs 4–6) to a bivariate view of fitness given in Fig 7A. This figure describes the function that links fitness to the underlying age-specific phenotypes at early and late ages (the *x* and *y* axes, respectively). A bivariate fitness optimum is arbitrarily set at the (0,0) phenotype. When viewed from the perspective, directional selection is proportional to the vector that finds the steepest fitness gradient at the point of the bivariate mean. Over a single generation, the population mean will change in both dimensions according to the effects of natural selection and new mutations. These processes can have effects on the phenotypes that act in concert or in antagonism, but an evolutionary equilibrium is found only when these causes of phenotypic change are equal and opposite.

Let us consider first the case when mutations tend to have a negative effect on both phenotypes. We can begin with an example of a non-aging population where both age-specific phenotypes are above the optima. This corresponds to population “1-” in Fig 7A. In this case, directional selection will cause the phenotypes to decrease (as this will increase fitness), and the effect of mutations will move the population in the same general direction. The population will quickly approach the optimum phenotypic values, but recurrent mutations will eventually push the age-specific phenotypes into values that are suboptimal for both ages, at which point selection will now favor phenotype increases. This process is illustrated by the arrow leading from “1-.” An equilibrium will be found at the point where increases caused by selection exactly cancel decreases caused by mutation. Recall that the changes due to mutation must be the same at both ages; this requires that selection must also be equal at equilibrium. This equilibrium point (indicated by the population with the dot at its center; Fig 7A) resides at a point where the ratio of selection gradients for early-age vital rates to late-age vital rates is equal to the ratio of vital rate gradients for late-age phenotypes to early-age phenotypes. This condition is met only when the late-age phenotype is further removed from its optimum than the early-age phenotype. This defines senescence, and because the late-age displacement is negative, this is hypofunction. This corresponds to the univariate perspective on MA and hypofunction illustrated in Fig 7A. Note that the starting point makes no difference to the location of the equilibrium. We had imagined that a non-aging population began with equally above optimal age-specific phenotypes, but we could just as easily imagine them as equally below optimal. In this case (population 2-), selection and mutation act in antagonism from the outset, the evolved change is smaller, but the equilibrium point is the same as before. The process is like that above if we assume instead that mutations tend to increase both age-specific phenotypes. However, regardless of whether our non-aging population begin with below optimal (2+) or above optimal (1+) phenotypes, the evolutionary equilibrium is found at the same phenotypes that describe senescence by hyperfunction (consistent with Fig 7B).

In the previous model (MA), new mutations were assumed to have effects on phenotypes at one age that were independent of effects on the other age. This allowed the age-specific phenotypes to evolve independent of each other. Viewed from the bivariate perspective provided in Fig 7A, natural selection was free to drive the population means directly up the fitness gradient in the direction of the global maximum. However, AP models assume that single alleles have effects on traits at multiple ages, and these effects have opposing effects on the vital rates at early and late ages. The effects on the age-specific phenotypes are contextual because the relationship between changes to phenotypes and, changes to vital rates depend upon where the population phenotypic means are located relative to their optima. For example, an allele that increases fitness and senescence by increasing age-specific phenotypes at both early and old ages can be qualify as an “AP” allele if that increase is advantageous when young and deleterious when old (Fig 7B). The optimality model implies that alleles with correlated effects at the 2 ages can be AP when one of 2 conditions are met: the age-specific phenotype means are on the same side of their optima and, the alleles have opposing effects on the phenotype; or the means are on different sides of their optima and, the alleles have effects on the phenotype that are in the same direction. We will demonstrate that both can lead to the evolution of senescence by either hypofunction or hyperfunction.

We consider first the former scenario. As before, we assume an initially non-aging population. The population mean can either begin equally below optimal for both age-specific phenotypes or equally above optimal (Fig 7B, bottom-left and top-right distributions). The population means are represented by dots, and because the allelic effects are highly positively correlated across the age-specific phenotypes, the joint distribution of genetic values that underly the population are depicted as being highly eccentric. In both cases, natural selection is not free to drive the population directly up the fitness gradients because very little genetic variation exists in that direction (there are no single alleles that can decrease or increase both phenotypes). Instead, the highly correlated allelic effects will constrain evolution to proceed along the axis of these distributions. For the case where both age-specific phenotypes begin above optimal, the population will find a position that maximizes fitness in obtainable parameter space that is near optimal for the early phenotype but above optimal for the late phenotype (hyperfunction). When both age-specific phenotypes are initially below optimal, selection will drive the population towards senescence via hypofunction.

If the population instead begins with one below optimal and one above optimal age-specific phenotype, and the alleles have the same effects on both (Fig 7B, bottom-right and top-left distributions), then the evolution of the phenotypes are constrained along axes that are orthogonal to those in the previous example. As before, the maximum fitness that is obtainable is located at age-specific phenotypes that demonstrate senescence. In this latter scenario, hyperfunction evolves from populations which begin with below-optimal early phenotypes and above-optimal late phenotypes. Hypofunction evolves from populations with the opposite initial configurations.

## Distinguishing between the DST and DTA models in empirical studies

The utility of any theory or hypothesis, evolutionary or otherwise, for our understanding of biological processes rests on its ability to provide clear and empirically testable predictions. It is important to note that both DST and DTA mechanisms may contribute to the evolution of aging. They may be of different importance for different pathways or phenotypes underpinning demographic aging and for different species depending on their ecological niche and evolutionary history. They could also conceivably operate side-by-side on the same aging pathway if they have independent effects on vital rates indirectly via resource allocation trade-offs and via independent effects that do not involve trade-offs with maintenance functions. In Table 1, we summarize some key assumptions and predictions of the 2 models. While the DST falls under the umbrella of AP, we have argued that the DTA is a broad suite of evolutionary models which can encompass the full range of age-specific genetic architectures covered by MA and AP models. For this reason, antagonistic age-specific genetic effects on vital rates predicted by AP are thus a shared prediction of both the DST and the DTA models. Thankfully, the 2 models do make some distinct predictions (Table 1). They also offer different perspectives of the possible uncoupling between growth and reproduction on one hand and aging on the other [55].

Most of the empirical evidence presented to date in support of the DST relates to trade-offs between traits associated with early- versus late-life vital rates, and this has much in common with the types of evidence and studies that are offered as support for AP in general (e.g., trade-offs between fitness components expressed at different ages [41,56]). Experimental manipulations of growth or reproductive effort, which impact lifespan or demographic aging in an antagonistic fashion, are often presented as supporting the DST [57,58]. However, artificially increased reproductive effort could result in an accelerated mortality rate in the absence of an increase in physiological damage [59], for example, by increasing exposure to predators or pathogens [60]. A growing number of observational long-term studies of animals in the wild have also documented negative correlations between individual early-life performance and rates of demographic senescence, and these are typically interpreted as supporting the DST (see [61] for a review and [14,62,63] for specific examples invoking the DST). The results of these studies are consistent with broad predictions emerging from the DST, specifically the existence of trade-offs between early- and late-life fitness components, even in eusocial species where fecundity and longevity can, at least to some extent, be uncoupled (see [4] for a specific discussion on this topic). Evidence for AP-like gene action or phenotypic early- versus late-life trade-offs are potentially consistent with both the DTA and the DST. This could be the result of those individuals showing reduced allocation of limited resources to maintenance leading to faster accumulation of damage (DST) or due to among-individual variation in expression of a gene or genes which enhance reproductive output in early life but lead to some form of physiological dysregulation later in life (DTA). As these studies neither explicitly demonstrate resource allocation (as in [64]) nor address the specific physiological mechanisms involved in such putative trade-offs, it is difficult to consider them as providing strong support for the DST while excluding other potential mechanisms (e.g., DTA-like mechanisms) [26].

## The DST and DTA as an evolutionary framework for the hallmarks of aging

Given the importance of accepted hallmarks of aging in biogerontology, a critical question beyond the role of both the DST and the DTA in early- versus late-life trade-offs remains whether and how particular hallmarks might have evolved to influence aging under these 2 models. One of the most widely discussed of the currently accepted hallmarks is “deregulated nutrient sensing.” This hallmark refers principally to clear evidence that suppressing the insulin/IGF-1 signaling (IIS) pathways, either genetically, pharmacologically, or via some forms of dietary restriction that can be underpinned in part by the IIS pathway, extends lifespan and retards aging in taxonomically disparate model organisms in the laboratory [65]. A DST-like model has been proposed to explain the evolution of the dietary restriction response [66]. Here, low food conditions are hypothesized to select for a greater allocation of limited resources towards maintenance, as successful reproduction is considered unlikely, and the best strategy is to “wait it out” until conditions improve and more resources can be obtained. The dietary restriction response is framed as an adaptive plastic response to varying resource availability. However, there is currently little direct empirical support for this mechanism, and assumptions of the model have recently been criticized [67]. Resource allocation is rarely directly measured, but 1 study using nitrogen isotope tracking in crickets (*Romalea microptera*) found no evidence that dietary restriction altered nutrient allocation [68].

There is growing support from studies of *Caenorhabditis elegans* that IIS pathways may support a core prediction of the DTA: that their role in aging can be delayed or reduced by suppressing or increasing their expression without a cost to reproduction (Table 1) [69–71]. This argues against a role for resource allocation trade-offs in observed variation in lifespan and aging [69,72,73]. Dillin and colleagues [70] showed that down-regulation of *daf-2* expression across the entire lifespan reduces reproduction, but that this cost can be avoided by limiting down-regulation to adulthood. Later work confirmed these findings for both mated and self-fertilizing modes of reproduction [71] and further demonstrated that adulthood-only *daf-2* knockdown increases lifespan without reduction in parental or offspring vital rates under both benign stable [71], stressful [74], and fluctuating environments [55]. Similarly, Ezcurra and colleagues [75] observed that intestinal atrophy and accumulation of lipoproteins were an important senescence-related pathology in hermaphrodite worms. They showed that this is driven by continued yolk production in adult worms; the worms convert intestinal biomass to yolk, driving intestinal atrophy and increasing late-life mortality [75]. Reduction in intestinal atrophy is associated with increased lifespan, while enhancing intestine-to-yolk conversion via IIS pathways reduces lifespan but, importantly, seems to increase fecundity in early adulthood. While other pathologies of aging have been putatively linked to hyperfunction in *C*. *elegans* [47], this offers arguably one of the clearest examples for a hyperfunction-like mechanism of the DTA involved in aging [75]. The emergent consensus on work with laboratory *C*. *elegans* does suggest that one key hallmark of aging, deregulated nutrient sensing, may have evolved through DTA.

Telomere attrition is another hallmark of aging [8] that has been related to the DST. Among the multiple hypotheses proposed to explain the evolution and function of telomere dynamics (see [76] for a review), it has been suggested that telomere maintenance could mediate the trade-off between growth and reproductive effort during early life and demographic aging later on [77]. Support for this hypothesis would require that: fast growth, and to some extent associated high reproductive effort during early life, are associated with a higher rate of telomere attrition [78,79], which might be expected due to greater cell proliferation rates or elevated reactive oxygen species production in faster growing individuals; and a causal relationship exists between shorter telomeres and increased late-life mortality risk [14] which, despite the evidence of association between telomere length and mortality risk [80], remains to be established [81]. Under our framework, an AP allele (or a set of alleles) could thus govern the allocation of resources toward growth and reproduction on one hand or telomere maintenance on the other, in line with the DST (Fig 2; see also [82]).

However, we would argue that additional conditions would need to be met before accepting telomere attrition as meeting the conditions of the DST. First, the energy requirement for maintaining telomere sequences above a critical length needs to be evidenced and quantified, as the energetically costly nature of somatic maintenance is a key distinction between the DST and the DTA (Table 1). The putative key role of resources in telomere maintenance has recently been put forward by the “metabolic telomere attrition hypothesis,” which proposes that energetic shortage might increase telomere attrition through a physiological cascade involving metabolic mediators such as glucocorticoids or AMP-activated protein kinase [83]. Current empirical support for such a scenario is principally limited to correlative studies linking environmental harshness and shortening telomeres [84]. The most convincing evidence comes from a food supplementation experiment performed in free-living edible dormice (*Glis glis*), in which food-supplemented individuals in a natural period of food shortage display almost no telomere loss compared with non-food-supplemented dormice [85]. Second, if the costly nature of telomere maintenance were to be established, experimental approaches would need to demonstrate that selection for an increased allocation towards growth or reproductive effort happens at the expense to an allocation towards the broad telomeric maintenance component, or vice-versa (Fig 2). However, several lines of evidence suggest that telomerase repression in somatic cells is adaptive, as short telomeres buffer the risk of cell proliferation once a cell becomes malignant (see [86,87] for reviews). The relative allocation of resources towards telomere maintenance mechanisms, such as telomerase activation (at the expense of an allocation towards growth and reproduction), and its benefits in terms of fitness should be largely modulated by the balance between cancer risk and aging, which itself depends on both local conditions and species life history [87]. Therefore, even if telomerase expression buffers biological aging [88], natural selection could favor, under high cancer risk, the allocation of resources towards DNA repair mechanisms other than telomere maintenance. Should this be demonstrated in the future, the role of telomere maintenance in the DST framework would most likely differ both between species and populations.

We are not aware of any suggestion in the literature that telomere attrition as a mechanism of aging might have evolved under the DTA, but this should not be discounted and could be empirically tested. For example, if suppression of telomerase in somatic tissues and associated progressive telomere shortening has been selected for in larger organisms to reduce the risk of displaying cancer early in life, weak selection in late life could result in suboptimally short telomeres across cell types in old age, which could trigger widespread cellular senescence and physiological dysfunction. This process would predict that experimentally enhancing telomere length in later life, without any manipulation of early life telomere length, would reduce aging.

Although full consideration of evidence across all of the 12 hallmarks of aging put forward by Lopez-Otin and colleagues [8] is beyond the scope of this work, dysregulated nutrient-sensing pathways and telomere attrition provide 2 interesting and exciting examples where theoretical and empirical research has begun to align these hallmarks with the DST and the DTA. Careful consideration of the predictions of the models alongside appropriate experimental design can illuminate the processes through which hallmarks of aging evolved.

## Conclusion: Towards an integrative evolutionary theory of aging

In this Essay, we proposed that our understanding of the biology of aging can be improved and progress in this field accelerated if we explicitly consider candidate mechanisms underpinning the aging process (such as the hallmarks of aging) within a broader framework of well-developed evolutionary theories. In a bid to advance the integration of biogerontology and evolutionary biology, we have attempted to frame 2 physiologically explicit theories of aging—the DST and the DTA—within the classical evolutionary genetic theories of aging, MA and AP. This requires the introduction of intermediate levels of the hierarchy between genes and vital rates. We hope this framework helps to highlight the core conceptual differences between the DST and the DTA and their relationship with the genetic theories, which we feel has been oversimplified in the literature. Moreover, we hope we have started to lay foundations for a more flexible and general framework that incorporates key mechanisms underpinning aging to further unravel how these complex processes evolved under natural selection and shape the diversity of aging patterns in the living world [10,11,89]. We emphasize that progress in holistic understanding of the biology of aging requires linking mechanistic approaches focusing on hallmarks with physiological and genetic theories of aging. To do so, mechanistic advances should be complemented with accurate estimates of the vital rates and, ultimately, Darwinian fitness, while evolutionary ecology studies should identify physiological mechanisms of aging that underlie putative genetic trade-offs and constraints [90].

The literature purporting to test the ideas of the DST and the DTA is fast growing and fascinating. However, a lack of clarity about the specifics of the model being tested and its prediction haunts most papers that invoke these 2 theories. Going forward, we caution researchers to be as explicit as possible about the kind of evolutionary model they wish to test in relation to the phenotype they are measuring. This will result in bridging the gap between molecular and applied biogerontology on one hand, and evolutionary biology and ecology on the other hand. Crucially, this will allow us to understand the relative importance of the DST and the DTA in the evolution of aging and will help to guide the efforts aimed at improving healthy aging without adverse effects.

Empirical studies focusing on the hallmarks of aging often seek to identify potential adverse effects associated with maximizing healthy aging. Decelerated aging and lifespan extension are often associated with reduced development, growth, or reproduction, which leads to invocation of the DST via allocation trade-offs in resource-limited environments. However, some studies do not find direct phenotypic trade-offs [91,92], which sometimes leads to the rejection of the gene-centric ETA. Neither approach is warranted because trade-offs between life-history traits that affect early- and late-life performance do not necessarily involve resource allocation and are also predicted by the DTA. Furthermore, the absence of early versus late reproduction or survival trade-offs is predicted under MA models. By re-framing the DTA in the context of an optimizing selection model, we have shown how it allows suboptimal late-life gene expression and phenotypes to evolve via MA processes, with no requirement for trade-offs or cross-age genetic correlations. Although some distinct and testable predictions for the DST and the DTA do emerge (Table 1), what is now required is a better understanding of how specific hallmarks of aging might fit into these evolutionary models. For example, recent work perturbing gene expression in key nutrient-sensing pathways suggests that the dysregulation of such pathways in later life may have evolved via the DST and/or the DTA [55,67]. The paths in the hierarchical models illustrated here (Figs 2, 5, and 6) can all be estimated empirically to address which model best reflects age-specific variation in each pathway, process, or phenotype. Further work to estimate the direction and magnitude of the pathways between genes and vital rates are pivotal to bridge evolutionary biology and biogerontology. We argue that efforts to do this will yield important insights into how and why hallmarks of aging vary in their importance across tissues, individuals, and species.

## Abbreviations

AP: antagonistic pleiotropy
DST: disposable soma theory
DTA: developmental theory of aging
ETA: evolutionary theory of aging
IIS: insulin/IGF-1 signaling
MA: mutation accumulation
TOR: target of rapamycin
